# Progressive Cerebellar Dysfunction, Pituitary Insufficiency, and Severe Skeletal Fragility in Adult Survivorship of Childhood Multisystem Langerhans Cell Histiocytosis: A Case Report

**DOI:** 10.1002/ccr3.73261

**Published:** 2026-07-29

**Authors:** Suhaib Alnahar, Alaa Senjab, Sara Gharib, Zena Gharib, Lama Hadid

**Affiliations:** ^1^ Faculty of Medicine Damascus University Damascus Syrian Arab Republic; ^2^ National University Hospital Damascus Syrian Arab Republic

**Keywords:** central diabetes insipidus, central hypogonadism, cerebellar dysfunction, hypopituitarism, Langerhans cell histiocytosis, secondary osteoporosis

## Abstract

Childhood‐onset multisystem Langerhans cell histiocytosis can lead to delayed adult morbidity involving the neurologic, hypothalamic–pituitary, and skeletal systems. Progressive cerebellar dysfunction, chronic pituitary insufficiency, and severe skeletal fragility may emerge years after apparent disease control, underscoring the need for prolonged multidisciplinary follow‐up.

## 1 Introduction

Langerhans cell histiocytosis (LCH) is a rare and heterogeneous disorder that predominantly affects children and is characterized by the clonal proliferation of myeloid precursor cells that differentiate into CD1a^+^/CD207^+^ Langerhans cells within lesional tissue [1, 2, 3]. Its overall annual incidence is estimated at one to two cases per million, with substantially fewer cases reported in adults [2]. Although multisystem involvement is relatively common in pediatric disease, reports describing the long‐term consequences of childhood‐onset multisystem LCH in adulthood remain uncommon and incompletely characterized in the literature [4].

Among the extracutaneous manifestations of LCH, hypothalamic–pituitary region involvement represents one of the most clinically important sequelae, affecting approximately 5%–50% of patients, especially those with multifocal disease [5]. In parallel, the skeleton is one of the most frequently involved organ systems, together with the skin [6]. While many osseous lesions undergo spontaneous healing and remodeling, a substantial proportion of patients develop permanent orthopedic morbidity, even in single‐system skeletal disease [7]. Long‐bone lesions most commonly involve the femur, followed by the humerus and tibia, whereas spinal disease often presents with osteolytic vertebral lesions that may progress to vertebra plana and structural deformity [8]. In addition, bone metabolism in LCH may be adversely affected not only by prior therapies such as glucocorticoids but also by endogenous mechanisms, including anterior pituitary hormone deficiencies and inflammatory cytokine‐mediated bone loss [9].

Beyond endocrine and skeletal involvement, LCH may also affect the central nervous system and give rise to a neurodegenerative phenotype. In adults, progressive neurological manifestations such as cerebellar ataxia and dysarthria have been described, sometimes in association with cerebellar atrophy on longitudinal neuroimaging [10]. Central nervous system involvement in adult LCH may also affect the hypothalamic–pituitary axis and can result in diabetes insipidus and varying degrees of pituitary dysfunction [11].

However, reports describing the combined late neurologic, hypothalamic–pituitary, and skeletal sequelae of childhood‐onset multisystem LCH in a single adult survivor remain scarce, and their long‐term clinicoradiologic correlation is insufficiently documented.

Herein, we report an adult patient with childhood‐onset multisystem LCH who, nearly two decades after the initial diagnosis, developed a complex pattern of late sequelae characterized by progressive cerebellar dysfunction, chronic hypothalamic–pituitary involvement, and marked skeletal fragility. By presenting the long‐term clinical evolution together with structural hypothalamic–pituitary abnormalities on MRI and severe bone mineral density loss, this case highlights a rare convergence of persistent neurologic, endocrine, and skeletal morbidity in adulthood and underscores the need for prolonged multidisciplinary surveillance in survivors of pediatric multisystem disease.

## 2 Case History

A 30‐year‐old Arab man with childhood‐onset multisystem Langerhans cell histiocytosis (LCH) presented after a prolonged disease course spanning nearly two decades with gradually progressive neurologic and musculoskeletal symptoms. At his most recent admission, he reported imbalance, gait instability, dysarthria, and dysphagia. He also described a subjective sense of heaviness in one lower limb without objective weakness, as well as intermittent blurred vision, occasional tinnitus, and reduced libido. In parallel, he reported chronic mechanical back pain, predominantly lumbar with intermittent cervical discomfort, aggravated by prolonged standing and physical activity. His history was also notable for multiple fractures beginning in childhood, with additional low‐impact fractures during adulthood. He denied headache, seizures, sensory deficits, sphincter disturbances, and inflammatory joint symptoms such as swelling, morning stiffness, or erythema.

The patient had originally been diagnosed with multisystem LCH at 10 years of age and had been treated with vinblastine and prednisone according to standard treatment protocols. During subsequent follow‐up, he developed hypothalamic–pituitary involvement manifested by growth hormone deficiency and central diabetes insipidus with polyuria and polydipsia, for which replacement therapy was initiated. During puberty, testosterone replacement therapy was started for central hypogonadism but was later discontinued in 2019.

On admission, his body mass index was 17.5 kg/m^2^. He was hemodynamically stable, alert, oriented, and not in acute distress. Neurologic examination showed preserved muscle strength (5/5) in all extremities. Cerebellar testing revealed impaired coordination on finger‐to‐nose and heel‐to‐shin testing. Romberg testing was positive, and gait assessment demonstrated a broad‐based gait pattern. Musculoskeletal examination showed mild tenderness over the lower lumbar spine without deformity, while peripheral joints were unremarkable. Mild hepatomegaly was noted on abdominal examination.

## 3 Investigations and Treatment

Given the progressive dysarthria, gait instability, broad‐based gait, and impaired cerebellar coordination in a patient with longstanding multisystem LCH, the neurologic findings were diagnosed as progressive cerebellar dysfunction representing late neurologic sequelae of LCH rather than LCH‐associated neurodegeneration. Concomitant hypothalamic–pituitary dysfunction was supported by the established history of growth hormone deficiency, central diabetes insipidus, and central hypogonadism. In addition, the combination of chronic back pain, recurrent low‐trauma fractures, and markedly reduced bone mineral density indicated severe secondary skeletal fragility. Inflammatory joint disease was considered unlikely because of the absence of joint swelling, morning stiffness, or erythema. No focal motor weakness or sensory deficit was identified on neurologic examination.

Laboratory evaluation demonstrated mild normocytic anemia, with a hemoglobin level of 12.8 g/dL. Endocrine assessment revealed a low‐normal testosterone level of 280 ng/dL with inappropriately low‐normal luteinizing hormone (2.0 mIU/mL) and follicle‐stimulating hormone (1.24 mIU/mL), supporting central hypogonadism. Additional laboratory parameters are summarized in (Table 1).

**TABLE 1:** Laboratory findings at the most recent admission.

Parameter	Value	Unit	Reference range
(Hb)	12.8	g/dL	13.5–17.5
Hematocrit(Hct)	39	%	41–50
Mean corpuscular volume (MCV)	82	fL	80–100
Erythrocyte sedimentation rate (ESR)	8	mm/h	0–15
Lactate dehydrogenase (LDH)	108	U/L	135–225
Alkaline phosphatase (ALP)	52	U/L	44–147
Calcium (Ca^2+^)	9.2	mg/dL	8.5–10.2
Testosterone	280	ng/dL	270–1070
Luteinizing hormone (LH)	2.0	mIU/mL	1.7–8.6
Follicle‐stimulating hormone (FSH)	1.24	mIU/mL	1.5–12.4
Serum iron (Fe)	82	μg/dL	65–175
Total iron‐binding capacity (TIBC)	280	μg/dL	250–450
Transferrin saturation	22	%	20–50

*Note:* Hematologic, inflammatory, biochemical, endocrine, and iron profile parameters measured during the patient's most recent admission, with corresponding reference ranges and units.

Abbreviations: ALP, alkaline phosphatase; Ca^2+^, calcium; ESR, erythrocyte sedimentation rate; Fe, serum iron; FSH, follicle‐stimulating hormone; Hb, hemoglobin; Hct, hematocrit; LDH, lactate dehydrogenase; LH, luteinizing hormone; MCV, mean corpuscular volume; TIBC, total iron‐binding capacity.

**FIGURE 1:**
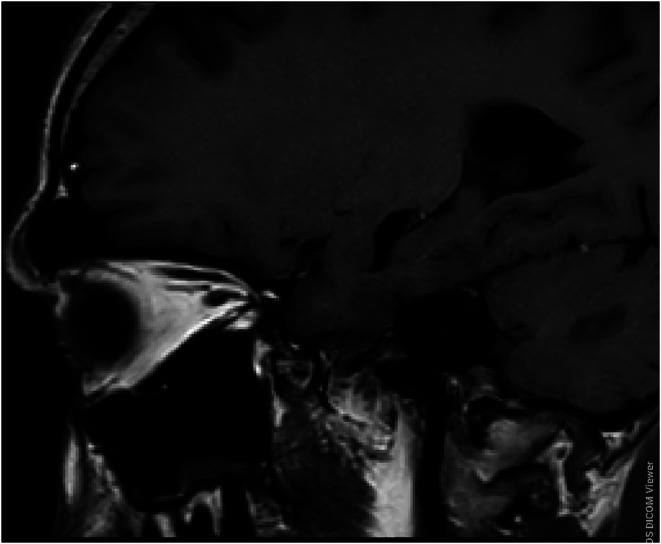
Sagittal pituitary magnetic resonance image demonstrating structural abnormalities of the hypothalamic–pituitary axis, including thinning of the pituitary stalk and a small anterior pituitary gland. The normal posterior pituitary bright spot is not identified.

Pituitary magnetic resonance imaging was performed using T1‐ and T2‐weighted sequences before and after contrast administration in sagittal, coronal, and axial planes. The examination demonstrated thinning of the pituitary stalk and marked reduction in the size of the anterior pituitary gland, with a maximal height of approximately 3 mm. The normal T1 hyperintense posterior pituitary bright spot was absent. The hypothalamus and mammillary bodies showed normal signal characteristics. The cerebral hemispheres, basal ganglia, and corpus callosum were unremarkable, with no abnormal signal changes. The cerebellum, brainstem, fourth ventricle, cerebellopontine angles, and craniocervical junction were also within normal limits, without focal signal abnormalities. These findings supported chronic structural hypothalamic–pituitary involvement, while the absence of characteristic abnormalities in the cerebellum, basal ganglia, or brainstem did not support confirmed neurodegenerative CNS‐LCH (Figure 1).

**FIGURE 2:**
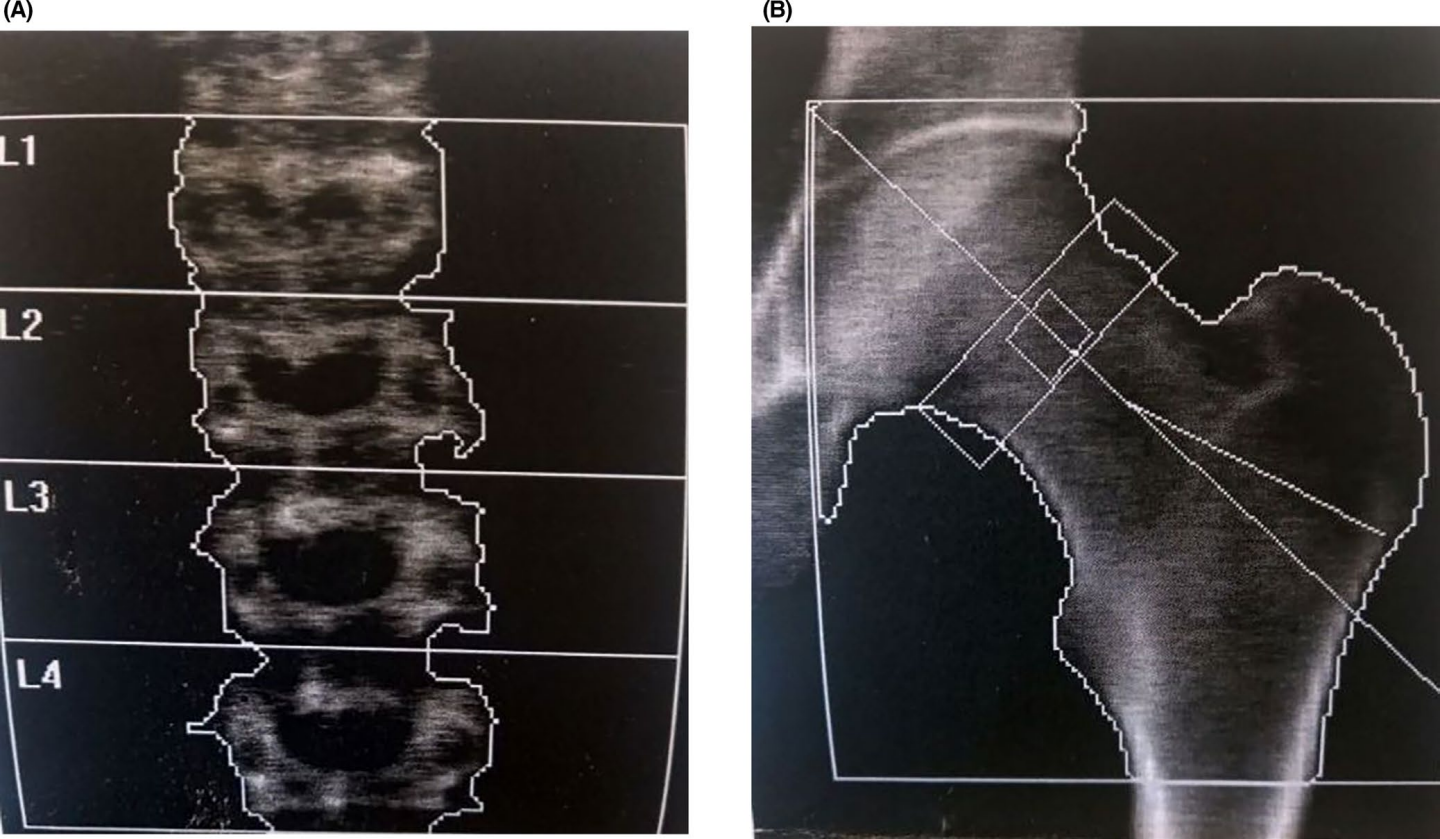
Dual‐energy X‐ray absorptiometry (DXA) showing severe reduction in bone mineral density. (A) Lumbar spine assessment (L1–L4) demonstrating markedly decreased bone mineral density. (B) Left hip assessment demonstrating markedly decreased total hip and femoral neck bone mineral density.

Bone mineral density was assessed using dual‐energy X‐ray absorptiometry (DXA). Lumbar spine assessment (L1–L4) demonstrated markedly reduced bone mineral density, with a total BMD of 0.522 g/cm^2^, corresponding to a T‐score of −5.2 and a Z‐score of −5.2. Assessment of the left hip also revealed markedly reduced bone mineral density, with a total hip BMD of 0.537 g/cm^2^ (T‐score, −3.3; Z‐score, −3.2) and a femoral neck BMD of 0.471 g/cm^2^ (T‐score, −3.4; Z‐score, −3.2). In the context of chronic endocrine dysfunction and recurrent low‐trauma fractures, these findings were consistent with severe secondary skeletal fragility (Figure 2A,B).

At the time of the current evaluation, the patient remained on hormone replacement therapy, anti‐osteoporotic treatment, and vitamin D supplementation. No major therapeutic modifications were made during the current assessment.

## 4 Discussion

This case illustrates the long‐term multisystem burden that can follow childhood‐onset Langerhans cell histiocytosis (LCH), because our patient reached adulthood with progressive neurologic dysfunction, persistent hypothalamic–pituitary disease, and profound skeletal fragility after nearly two decades of illness, a pattern that aligns with cohort data showing that permanent consequences are common after LCH, particularly in multisystem disease and in patients with reactivations [12, 13].

In the long‐term pediatric cohort analyzed by Chow et al., sequelae were documented in 56% of patients, with orthopedic abnormalities, diabetes insipidus, growth impairment, neurologic deficits, and anterior pituitary hormone deficiency all represented, and neurologic sequelae could emerge even 10 years after the initial diagnosis, which strongly supports the biological plausibility of our patient's delayed adult morbidity [12]. Our patient's course is therefore consistent with the concept that LCH survivorship is not defined merely by control of the initial lesions, but by an extended risk of irreversible endocrine, skeletal, and neurologic complications that may continue to evolve long after the acute phase of the disease has apparently stabilized [12, 14].

The clinical significance of this case lies less in any isolated manifestation and more in the convergence of three major late domains of injury, neurologic, hypothalamic–pituitary, and skeletal, in a single adult survivor of childhood multisystem LCH [12, 13, 15, 16].

The neurologic component of our patient's presentation is particularly noteworthy, as his progressive imbalance, broad‐based gait, dysarthria, dysphagia, and impaired cerebellar coordination closely resemble the clinical manifestations described within the neurodegenerative CNS‐LCH spectrum, including ataxia, dysarthria, dysmetria, tremor, gait disturbance, and later neurobehavioral impairment [14]. McClain et al. further characterized LCH‐associated neurodegeneration as a delayed complication that may emerge decades after apparent control of systemic disease and is typically associated with T2/FLAIR abnormalities involving the cerebellum, basal ganglia, and brainstem. Neuropathologic studies have also demonstrated BRAFV600E‐positive myeloid/monocytic infiltrates, osteopontin expression, gliosis, and active demyelination, supporting an ongoing biologically active process rather than a purely static residual injury [15]. In our patient, however, the absence of characteristic cerebellar or brainstem abnormalities on the available imaging does not support confirmed LCH‐associated neurodegeneration. Therefore, his progressive cerebellar dysfunction is best interpreted as a late neurologic sequela of LCH with clinical overlap with the neurodegenerative CNS‐LCH spectrum [12, 14, 15].

This distinction is further supported by the fact that central diabetes insipidus and CNS‐risk disease are repeatedly associated with subsequent CNS morbidity. Our patient had longstanding central diabetes insipidus, placing him within a clinically high‐risk framework [12, 14, 17].

Chronic central diabetes insipidus, growth hormone deficiency, and central hypogonadism together in our patient fit well within the established spectrum of hypothalamic–pituitary LCH, but the structural imaging pattern in our patient is more unusual than the commonly reported active sellar phenotype [17, 18]. In the HEROS cohort of patients with hypothalamic–pituitary LCH, 69% had arginine vasopressin deficiency at diagnosis, 42% had central hypogonadism, and 73% had posterior pituitary or stalk abnormalities on MRI, while additional hormonal deficits could still appear during long‐term follow‐up, underscoring that endocrine injury may remain dynamic over many years [13].

By contrast, many isolated hypothalamic–pituitary case reports describe enlarged or nodular pituitary stalks, homogeneous enhancement, or suprasellar masses, as in the reports by Zhou et al. and Chauhan et al., whereas our patient showed stalk thinning, a very small anterior pituitary gland, and loss of the posterior pituitary bright spot, which together suggest chronic structural aftermath rather than active infiltrative expansion [18, 19].

Importantly, the adult case reported by Lian et al. provides a partial radiologic parallel to our patient because that patient also had marked pituitary volume loss with an empty‐sella‐like appearance and infundibular abnormality, indicating that hypothalamic–pituitary LCH can evolve beyond the classic thickened‐stalk pattern into chronic atrophic structural change [20].

The persistence of replacement therapy in our patient is therefore unsurprising, because the literature consistently shows that once pituitary damage is established, endocrine recovery is limited and systemic therapy usually exerts only modest benefit on established hormonal deficits [13, 18].

The skeletal involvement in our patient was particularly severe and extended beyond the focal osseous lesions classically associated with LCH. At 30 years of age, he had recurrent fractures, including low‐trauma fractures during adulthood, together with profoundly reduced bone mineral density. His lumbar spine Z‐score of −5.2 and hip Z‐scores of approximately −3.2 indicated bone mineral density markedly below the expected range for age. In this clinical context, the combination of markedly reduced age‐adjusted bone mineral density and recurrent fragility fractures supported severe secondary skeletal fragility rather than an isolated densitometric abnormality.

The pathogenesis of bone fragility in this patient was likely multifactorial. Longstanding growth hormone deficiency and central hypogonadism may have impaired the acquisition and maintenance of bone mass, while discontinuation of testosterone replacement may have further contributed to skeletal deterioration. His markedly low body mass index, previous exposure to prednisone, and possible reduction in physical activity related to progressive cerebellar dysfunction represented additional adverse factors. These patient‐specific contributors likely acted in combination with the direct effects of LCH on bone metabolism.

Makras et al. reported bone mineral density below the expected range for age in 20% of adults with LCH and demonstrated significantly lower lumbar spine Z‐scores in patients with active disease than in those with inactive disease and healthy controls [16]. The substantially greater reduction observed in our patient highlights the cumulative skeletal burden that may develop after childhood‐onset multisystem disease accompanied by persistent endocrine dysfunction. In addition, LCH‐related inflammatory pathways may promote skeletal loss through increased osteoclast activity mediated by cytokines such as IL‐1 and TNF‐α and dysregulation of the RANK/RANKL/OPG system. Glucocorticoid exposure, chemotherapy, and anterior pituitary hormone deficiencies may further impair bone remodeling and amplify fracture risk [21]. The severity of skeletal involvement in this case emphasizes the importance of long‐term bone surveillance, optimization of pituitary hormone replacement, serial assessment of bone mineral density, fracture‐risk reduction, and continued bone‐directed therapy in adult survivors of childhood LCH.

Taken together, this case is distinctive because it documents an uncommon adult end‐stage phenotype of childhood‐onset multisystem LCH in which progressive cerebellar dysfunction, chronic hypothalamic–pituitary insufficiency, and extreme skeletal fragility coexist, thereby reinforcing the need for prolonged multidisciplinary surveillance that is neurologic, endocrine, and bone‐focused rather than lesion‐focused alone.

## 5 Conclusion

Childhood‐onset Langerhans cell histiocytosis (LCH) can lead to long‐term, multisystem complications in adulthood. This case demonstrates how progressive cerebellar dysfunction, chronic endocrine insufficiency, and severe osteoporosis may emerge decades after initial diagnosis. Recognizing these delayed sequelae and ensuring structured, multidisciplinary follow‐up are crucial for optimizing outcomes and reducing morbidity in adult survivors of childhood LCH.

## Author Contributions

**Sara Gharib:** writing – original draft, investigation, methodology, validation. **Alaa Senjab:** project administration, writing – review and editing, writing – original draft, visualization, conceptualization. **Suhaib Alnahar:** conceptualization, data curation, writing – original draft, validation, investigation. **Zena Gharib:** writing – original draft, investigation, validation, methodology. **Lama Hadid:** supervision, writing – review and editing, investigation, validation.

## Funding

The authors have nothing to report.

## Ethics Statement

Institutional Review Board (IRB) approval is not required for de‐identified single case reports or case histories, in accordance with institutional policies.

## Consent

Written informed consent was obtained from the patient for publication and any accompanying images. A copy of the written consent is available for review by the Editor in Chief of this journal on request.

## Conflicts of Interest

The authors declare no conflicts of interest.

## Data Availability

Data sharing not applicable to this article as no datasets were generated or analyzed during the current study.
